# “Leading through Crisis”: A Systematic Review of Institutional Decision-Makers in Emergency Contexts

**DOI:** 10.3390/bs14060481

**Published:** 2024-06-06

**Authors:** Ivan D’Alessio, Alessandro Quaglieri, Jessica Burrai, Alessandra Pizzo, Emanuela Mari, Umberto Aitella, Giulia Lausi, Ginevra Tagliaferri, Pierluigi Cordellieri, Anna Maria Giannini, Clarissa Cricenti

**Affiliations:** Department of Psychology, Sapienza University of Rome, 00185 Rome, Italy; alessandro.quaglieri@uniroma1.it (A.Q.); jessica.burrai@uniroma1.it (J.B.); alessandra.pizzo@uniroma1.it (A.P.); e.mari@uniroma1.it (E.M.); aitella.1712631@studenti.uniroma1.it (U.A.); giulia.lausi@uniroma1.it (G.L.); ginevra.tagliaferri@uniroma1.it (G.T.); pierluigi.cordellieri@uniroma1.it (P.C.); annamaria.giannini@uniroma1.it (A.M.G.); clarissa.cricenti@uniroma1.it (C.C.)

**Keywords:** emergency decision making, emergency management, crisis response strategies, disaster preparedness strategies, emergency response tactics, catastrophe mitigation strategies, crisis management, cognitive psychology, decision psychology, cognition

## Abstract

This review aims to map studies on governmental and institutional decision-making processes in emergencies. The literature reveals various approaches used by governments in managing emergencies. Consequently, this article suggests the need for a systematic literature review to outline how institutional decision-makers operate during emergencies. To achieve this goal, the most widely used databases in psychological research were consulted, with a specific focus on selecting scientific articles. Subsequently, these studies were rigorously assessed for their relevance using a structured literature selection process following the PRISMA 2020 guidelines. At the conclusion of the review process, nine studies were identified, each suggesting different methods by which governments manage emergencies. This diversity arises because emergency decision-making processes must account for numerous variables that change depending on the type of crisis and the specific context. However, several critical aspects have emerged, such as the centrality of pre-disaster planning to improve intervention practices and methods, attention to information gaps that inevitably arise during an emergency, and the importance of streamlining and delegating decision-making to emergency responders in the field to counter the phenomenon of centralized decision-making that often hampers crucial interventions during emergencies.

## 1. Introduction

Natural disasters on a global scale are constant evidence that drives human beings in a continuous race towards the best way to manage problems resulting from sudden and unforeseen catastrophes. Mass disasters take different forms, ranging from natural and environmental hazards to large-scale incidents (e.g., epidemics, drastic economic fluctuations, terrorist attacks, sudden conflicts, and severe financial crises) [1]. A crisis can occur because of an unforeseeable event or an unforeseen consequence of an event considered to be a potential risk [2]. According to Eden and Matthews (1996), a crisis is defined as “any incident that poses a threat to human safety and/or causes damage to animals, buildings, equipment and systems” [3].

The characteristics of crises vary, and they involve a sudden and serious threat, a lack of time and resources, inadequate infrastructure to deal with them, and a high degree of uncertainty about their evolution. The decision-making environment in these cases is characterized by considerable complexity due to changing circumstances, limited reaction time, disparity of information, and inadequate protection of resources. This creates an urgent and substantial demand for Emergency Decision Making (EDM). In terms of decision-making goals, practitioners should set as their main objective the increased development of EDM skills for natural disasters to minimize the loss of life [4]. Sudden and unpredictable crisis events disrupt the “normal fabric of community life”, causing widespread panic and uncertainty among the population. In these critical moments, community leaders are tasked with responding to these extraordinary circumstances. A useful key to better understand these contexts is offered by complexity theory.

Complexity theory is defined in the literature, starting with systems theory, as a subset of all systems: a diverse and heterogeneous subset that is the basis of all novelty; a subset that manifests itself in biological, chemical, physical, social, technical, and economic domains; a subset that co-evolves with its environment and is self-organizing, emerging from multiple and continuous interactions [5]. Boulton and colleagues, in their book “Embracing Complexity: Strategic Perspectives for an Age of Turbulence,” summarize what they call “the central principle of complexity theory” and examine the topic of complexity in depth. Change, according to their perspective, takes the form of the interaction between “the detail and the variation” of each action. The continuous interaction between different individuals leads to a connection between actions and the environment that “provides the fuel for innovation, evolution, and learning” [6]. Over the past thirty years, as complexity theory developed, the applied subset of “complex problem solving” (CPS) [7,8,9,10] emerged in this field. This field of study aims to describe how people deal with complex, dynamic, and non-transparent situations. CPS is described in the scientific literature as the acquisition of knowledge and practical application of strategies and actions useful for achieving objectives of systems that contain many highly interconnected elements (for example, complex systems) [11]. CPS activities are situations that are defined as follows: dynamic because the properties that constitute complex problems are constantly changing; time-dependent because decisions must be made at the right time in relation to the demands of the context; and complex because most of the variables are not correlated with each other [12]. Complex problem-solving is closely related to naturalistic decision-making and dynamic decision-making. Naturalistic decision-making (NDM) is concerned with decision-making in real-life contexts, with the ambition of helping people develop skills more rapidly in response to the challenges they face daily. One of the significant features of the NDM field is that it explicitly seeks to understand how people handle complex tasks and environments. NDM examines phenomena in the context of their situations and uses this understanding to develop useful tools to support human decision-making processes [13]. The study of dynamic complexity helps to understand basic decision-making processes and aids in the identification of human limitations in decision-making in complex and dynamic tasks [14].

Dynamic decision-making (DDM) animates different contexts: military, medical, managerial, and many others often having some connection with the emergency domain [15,16]. In this sense, decisions require an increasing degree of complexity, are driven by a greater degree of uncertainty, and involve increasing time pressure that makes everything more difficult for the decision-maker in addition to rapidly changing circumstances. Common characteristics of complex decisions include sequential sampling of information, constant detection of change, monitoring of progress, resource estimation, portfolio management, emergency resource allocation, and many other activities. Disasters present significant obstacles for both affected communities and individuals in terms of preparing for, understanding, and responding to adverse events. They are characterized by unpredictability, a tendency to cause severe consequences, risks and time constraints, and depletion of existing resources. Overall, these factors undermine the effectiveness of established contingency plans [17]. Scholars have emphasized the need for an interactive relationship between policy and science to address the persistent and evolving challenges posed by changing risk scenarios [18]. In the literature, various research approaches seek to clarify how institutional decision-makers orient themselves in emergency and high-risk situations. Emergency decision-making represents a complex and evolving challenge due to the high levels of risk and uncertainty inherent in the emergency environment [19]. The urgency for quick decisions under pressure renders bureaucratic processes ineffective [20]. The demands for urgent decision-making and immediate response conflict with formal and time-consuming political procedures [21]. Over the past two decades, the European public has increasingly supported governmental involvement in decision-making at crucial moments [22,23,24]. The importance of developing accurate institutional theories and models is becoming an increasingly common theme in the literature on public policy and organization theory for efficient decision-making processes [25]. Several scholars have compared the different approaches to institutional theory, typically distinguishing “rational choice” institutionalism in political science and economics from the branch of institutionalism in sociology [26,27,28,29]. Decision-making in emergency management has been widely addressed by scholars in the field [30,31,32]. Traditional methods of public emergency management are characterized by policies based on centralization and hierarchy [33,34]. More recently, the importance of distributed and participatory approaches to decision-making by institutions in large-scale emergencies or crises has been emphasized [35]. It is important not to overlook the classification of problem types. In the literature, problems have been categorized as simple, complicated, and complex [36]. Simple problems are easily representable by humans and consist of well-understood essential elements. Complicated problems contain subsets of simple problems but cannot be reduced to them easily. Complex problems, while generalizable, tend to be unique and subject to numerous constraints, consisting of many variables [36].

A key element in crisis management is Command and control (C2) applications. C2 systems are applied in a variety of fields: from the military to public safety to civil protection in emergency and disaster management. Command and control encompass all planning and response functions and operations, integrating and harmonizing them. Command and control are how a commander recognizes what needs to be done and ensures that appropriate action is taken [37]. To deal with dynamic and complex scenarios in the best possible way, working practices have been consolidated using teams to mitigate the risk of failure from a strategic point of view. Thus, many efforts have been made in the scientific community to understand the knowledge, skills, and attitudes of teams operating in highly complex contexts, such as those of command-and-control teams [38].

The requirements for a C2 system that can provide a continuous response to a major crisis and offer a training environment to ensure preparedness are situational awareness of casualties and resources, response/communication coordination, information management/analysis, and simulation capabilities for predicting future scenarios. The bigger the disaster, the more important it is to have an effective C2 structure. Currently, it is the technological solutions that make unlimited disaster management possible. New technologies offer opportunities to improve and extend information retrieval, visualization, decision support, and communication within the crisis management domain. However, these supports cannot ignore the essential role of the human being in decision-making [39]. The C2 system may emerge through different mechanisms: it may take the form of a conscious command decision, an immediate reaction, or a procedure based on strict rules.

### 1.1. Emergency Decision-Making in Emergency Disasters

There are several interpretations of contingency decision-making theory, and their key points seem to overlap with each other. In general, emergency decision-making theory examines the decision-making processes that occur when people respond to a negative event and identifies the factors that predict response choices. K. Sweeny identified a model characterized by a three-stage process that individuals go through when facing a negative life event: in the first stage, individuals assess the severity of the negative event using various types of information; secondly, individuals determine response options; and thirdly, they evaluate response options before implementing their chosen responses [40]. On the other hand, Yu and colleagues (2011) emphasize how emergency decision-making can be outlined in six stages: problem definition, goal establishment, project design, project selection, organizational implementation, and feedback modification [41]. However, emergency decision-making involves several common elements: timely collection of relevant information, clear definition of emergency objectives, formulation of operational plans, execution, coordination, supervision, and tailored responses to unique circumstances [42,43,44].

In crises, emergency organization and communication patterns are severely compromised [45]. Social context, personal motivations, and automatic processing can influence individuals’ decision-making processes in the face of crises [40]. In emergency scenarios, decision-making may require sequential adjustments to align with the specific context in which decisions are made. From the perspective of decision-makers in Emergency Decision Making (EDM), it is crucial to involve multiple sectors in disaster prevention and mitigation efforts. Furthermore, an effective coordination of decisions plays a critical role in EDM, contributing to the creation of a highly centralized decision-making core and strong executive departments within organizations.

Several studies have examined how crisis and emergency scenarios are managed. Rawls and Turnquist (2012) point out how research on emergency operations management has increased in recent decades [46]. Alkandari and Al-Lozi (2017) and Oktari et al. (2020) explored how the strategic use of information can be applied in all phases of crisis management to confirm the benefits of knowledge use in reducing disaster impacts and improving resilience [47,48]. Hazaa et al. (2021) focused on the most crucial aspects and factors influencing a crisis [49]. Goldschmidt and Kumar (2016) analyzed cases of humanitarian aid and simultaneous emergency management to provide useful insights for practitioners [50].

### 1.2. Aim of the Study

Despite several studies having examined the decision-making processes of emergency managers during critical events, to the best of our knowledge, no reviews have assessed the analysis of models and decision-making strategies adopted by institutional emergency managers who find themselves making decisions in critical contexts (such as policymakers, civil protection, law enforcement, healthcare workers, volunteer responders, etc.). Our study aims to shed light on decision-making issues through an analysis of the phenomenon from multiple perspectives, examining the various methods and approaches taken by experts in this field.

The research question that inspired this review was as follows: “Are there any studies that have investigated decision-making processes in the field of emergency management, with particular reference to institutional decision-makers, in order to find and list useful data for future research in this area?”

Based on these premises, the purpose of our review was to (1) collect research data on models and strategies related to decision-making processes adopted by institutional decision-makers involved in managing and coping with emergencies, and (2) highlight the key characteristics of these decision-making methods regarding these phenomena by comparing data from various studies on the subject. The target population of this study includes all institutional decision-makers involved in emergency management who act whenever an emergency or critical event threatening public safety occurs.

## 2. Materials and Methods

The procedure involved the use of a systematic approach to the literature selection. This systematic literature selection enabled a structured search strategy, with clearly defined inclusion and exclusion criteria, promoting transparency and impartiality. This ultimately enhances reliability, as potential biases in the literature selection can be minimized and openly discussed [51]. The systematic literature review was conducted following the PRISMA methodology (Preferred Reporting Items for Systematic Reviews and Meta-Analyses) [52,53]. The study was registered in the “International Prospective Register of Systematic Reviews” (PROSPERO) in October 2023 (CRD42023469096), and the detailed protocol is available upon request.

### 2.1. Inclusion Criteria

**Figure 1:**
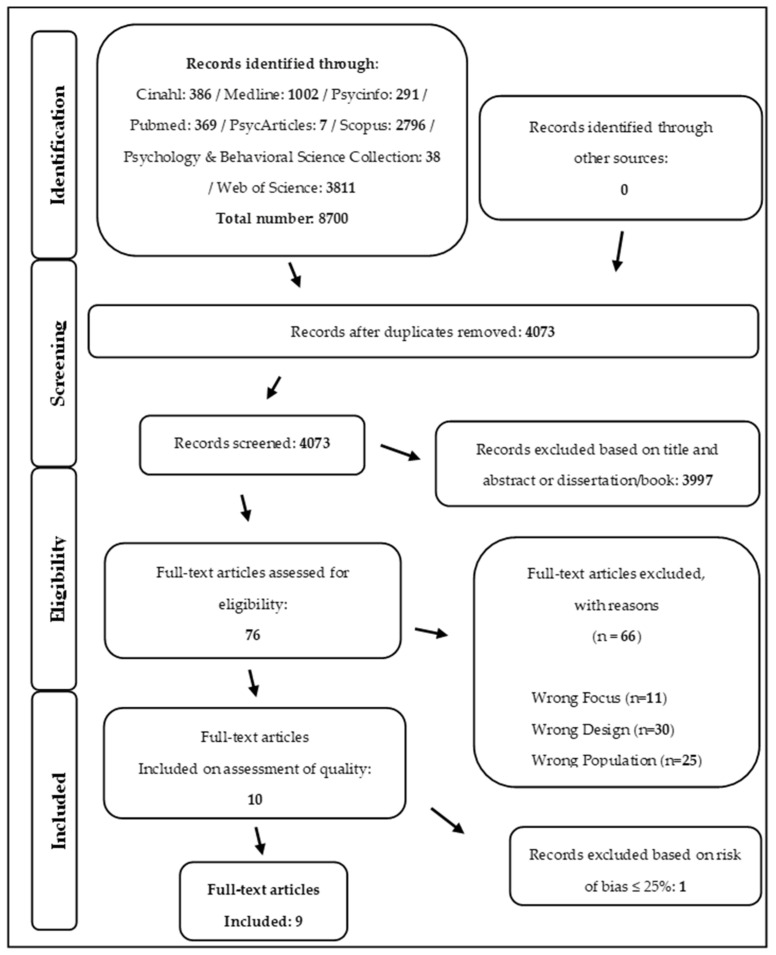
Flow diagram outlining the literature selection process according to PRISMA criteria.

To proceed with the investigation, we first defined our area of interest, namely, “Decision-making models and strategies adopted by institutional decision-makers in emergency contexts”. We then established the alphabetical string to be used in different scientific search engines. The research string for scientific database queries was constructed using the following Boolean operators: “(Decision Making Strateg * OR Decision-Making Process * OR Decision-Making Model *) AND (Emergenc* OR Disaster * OR Crisis OR Catastroph * OR Traged *)”. Subsequently, we downloaded results from different scientific search engines to consolidate them into a single Excel file. The scientific databases used included Scopus, Web of Science, PubMed, Medline, PsycInfo, PsycArticles, CINAHL, Psychology and Behavioral Science Collection. The inclusion criteria for selecting relevant studies were based on study type (qualitative/quantitative, longitudinal/cross-sectional, and cohort), population (institutional decision-makers, or government and/or political and/or military experts responsible for emergency management and decision-making process), focus (decision-making models and/or strategies in emergencies, risk, uncertainty, and/or catastrophe), and the presence of mass disasters or large-scale emergencies. Documents were excluded if their abstract addressed areas beyond emergency and disaster management, or if they were categorized as review articles, dissertations, technical reports, or white papers, or if they were not written in English. Only works published from early 2001 until the end of 2022 were considered to obtain more updated data in line with contemporary phenomena. At this point, we were divided into two groups to work blindly. Each group independently worked on the file containing the research results. Zotero software v. 6.0.9 was used as a tool for the analysis and cataloging of the literature. Out of the 8700 articles identified from various databases, 4073 articles of interest were identified after removing duplicates. Duplicate records were eliminated using Rayyan’s duplicate management function. Subsequently, during the selection of the 4073 identified articles, 3997 articles were excluded based on the analysis of the title and abstract. Two authors independently coded the screening of titles and abstracts. Works belonging to the categories of theses or books were also excluded. This left 76 articles; at this stage, we proceeded with reading the full text. From the full-text reading, 76 eligible articles were selected, while 66 were excluded. A total of 11 articles were excluded due to incorrect focus, 30 articles for incorrect design, and 25 articles for incorrect population inclusion criteria. At this point, only 10 out of the 76 articles met the inclusion criteria qualitatively. Of these 10 articles, the bias risk assessment led to the exclusion of an additional article, resulting in the final selection of 9 studies. Two researchers independently conducted reviews of the titles and abstracts of all articles. Both researchers were kept unaware of each other’s decisions. After every screening of 300 articles, any discrepancies were addressed through discussion until a consensus was reached. Where consensus was not reached, a third researcher was consulted. The same procedure was adopted to independently assess the full texts for article inclusion. Again, in case of disagreement, consensus was reached through discussion, and where necessary, consulting a third researcher. Mendeley Desktop reference management software v. 1.19.8 was used to facilitate the screening process. Subsequently, two different researchers than those previously involved conducted data extraction using a standardized form. To ensure consistency, these two researchers compared their work with a third researcher. Any discrepancies were resolved through discussions, aiming to reach a consensus. A schematic representation of this literature selection process can be seen in Figure 1. Blindly working researchers achieved the same results; at the end of each previously described phase, there was a comparison of the intermediate results obtained.

### 2.2. Risk of Bias Assessment

Two authors also performed a risk of bias assessment using two NIH quality assessment tools (https://www.nhlbi.nih.gov/, accessed on 28 September 2023). A third author performed a final review to ensure the appropriateness of the assessment procedure. A general quality rating assessment was performed to identify the general risk of bias amount: studies were rated as “good” if they showed ≥75% of positive answers to NIH tool questions (N = 3), they were rated as “fair” if they showed 50–75% of positive answers to NIH tool questions (N = 5), and they were rated as “poor” if they showed 25–50% of positive answers to NIH tool questions (N = 1). Studies reporting 25% of positive answers to the tool were rated as “very poor” and thus excluded (N = 1). Hence, 10 studies were reviewed (see Table 1). The articles selected at the end of the literature selection process are listed in Table 2.

## 3. Dataset and Results

The selected studies were mainly cross-sectional and observational cohort studies. The studies were conducted in different countries: a total of four articles were from the United States of America [54,55,57,61], two from the United Kingdom [54,63], one from the Republic of South Africa [54], one from Iran [58], two from Turkey [54,56], one from Pakistan [58], one from Iraq [59], and finally one from New Zealand [60]. Except for one study conducted in 2013, all other studies were conducted recently: one in 2017 [57], four in 2018 [54,55,56,60], one in 2019 [58], one in 2020 [59], and one in 2021 [63]. In most of the studies, mainly ad hoc questionnaires [54,55,63] or semi-structured interviews [56,57,59,60] were used to assess decision-making competencies; however, the GDMS Inventory [58] was used in one study and the WGCTA-S [61] was used in one study. All studies had as their sample decision-makers belonging to institutions that were responsible for emergency management at different levels: one study surveyed 15 government experts from the United States of America, the United Kingdom, the Republic of South Africa, Iran, and Turkey [54]; one study surveyed 231 public managers specialized in emergency management in the United States of America [55]; one study interviewed 23 emergency managers at the municipal level in the United States of America [57]; one study surveyed 268 emergency operations officers from the health, public safety, and civil protection sectors in Pakistan [58]; a study interviewed 54 local emergency managers in the United States of America [61]; a study surveyed 58 key decision-makers in emergency management in Turkey [56]; a study interviewed 185 emergency operations officers from the health, public safety, and civil protection sectors in the United Kingdom [63]; a study surveyed 15 crisis management experts in Iraq [59]; and finally, a study interviewed 30 crisis management experts from different organizations in New Zealand [60].

The inadequacy of communication, the difficulty in timely and accurate exchange of information, and the difficulty in accessing useful data during emergency management by institutional decision-makers were frequently encountered problems of particular importance [54,56,60,63]. Another issue reported was the lack of clear recognition of coordination responsibilities along with the lack of coordination of responses in crisis scenarios, aspects that often paralyze the smooth continuation of operations [54,60]. Adequate training of personnel employed at all levels in emergencies is a crucial factor in managing unforeseen accidental events. Poor emergency management skills on the part of institutional decision-makers stem precisely from a lack of adequate training [54,60]. Adequate education has helped prepare individuals to think critically, and these cognitive skills serve as a guide in emergencies in addition to the indispensable role of practice-based experience [54,61]. Institutional decision-makers seem to be more risk-averse when scenario outcomes are presented as gains than when the same outcomes are presented as losses, and it has also been found that many institutional decision-makers would disapprove more of an error of omission in the operational planning of an intervention than that of an error of commission in dealing with an emergency [55]. On the level of emotions, it was found that emotional intelligence moderates the relationship between operational stress and decision-making styles: in practice, institutional decision-makers with high emotional intelligence, instead of panicking and adopting ineffective decision-making strategies, remain focused and adopt effective decision-making styles despite being in very stressful situations [58].

Finally, the aspects most frequently considered and deemed central by government decision-makers regarding emergency decision-making were the presence of a multitude of people involved in a crisis, the continually changing conditions of an emergency scenario, the availability of multiple choice options to deal with a problem, the assumption of objective data when available, the existence of governance objectives, the evaluation of decision outcomes, the structure of the organization in charge of crisis management, the presence and impact of time constraints, the evaluation of all possible options available, the possibility of conversations with stakeholders, the presence of supportive decision-making experts, the focus on multiple problems at the same time, and decision-making based on heuristics [54].

## 4. Discussion

This study thoroughly examined emergency decision management (EDM) during disasters, focusing on a variety of factors and key points that emerge from the existing literature. The studies analyzed highlighted several challenges and issues faced by institutional decision-makers during emergency management, along with predictive factors and effective strategies for dealing with such situations. One of the main findings concerns inadequate communication and inefficient exchange of information during crises. This problem can compromise the timeliness and accuracy of decisions made during emergency management, highlighting the need to improve communication systems and access to useful data for institutional decision-makers. This is of fundamental importance because the correctness of the processed data and communication efficiency are at the basis of efficient complex problem-solving (CPS) and dynamic decision-making (DDM) systems. Although the importance of the adequate training of personnel involved in emergency management is an essential factor for a better response to critical moments, this is not always true. In fact, we must deal with the computational limits of human cognition in dynamic decision-making (DDM) [8,64,65,66]. Indeed, the natural limits of human cognition affect the ability to perfectly represent problems, often leading decision-makers to make mistakes, especially in complex problem-solving (CPS) and dynamic decision-making (DDM) [67]. To better handle complex situations, such as sudden emergency scenarios, it is necessary to adopt a systemic approach, considering the multiple components of a system and clearly and intelligibly representing all the relationships between the core of the complex problem and its connected components. This necessary methodology helps to overcome the limits of human cognition [66].

A lack of clarity in coordination responsibilities and poorly coordinated responses during crises are frequently reported problems. These factors can hinder the operational flow of emergency operations, underlining the importance of defining clear roles and responsibilities and promoting better collaboration between the parties involved. At the institutional and governmental level, the decision-making structure is structured in a pyramid fashion regardless of the context. This is the classic case of command and control (C2) structures. One should not think of such models as rigid and stereotypical, but rather a valid command and control architecture prevents and mitigates the problems arising from unclear attributions of responsibility, which, as it turns out, result in situations of poor coordination to the point, in some cases, of leading to decision-making immobility. Furthermore, it was found that emotional intelligence plays an important role in moderating the relation between operational stress and decision-making styles. Individuals with high emotional intelligence are better able to remain calm and adopt effective decision-making styles even under pressure, suggesting the importance of developing emotional competence in the context of emergency management. By working on the emotional aspects of institutional decision-makers, it is possible to develop protective factors in terms of vulnerability to operational stress and, above all, to better manage emotions during critical events in such a way that they do not negatively affect decision-making processes. Finally, governmental decision-makers have identified several central aspects concerning emergency decision-making, including the management of multiple-choice options, the evaluation of governance objectives, and the presence of time constraints. These factors influence decision-making during crises and underline the importance of considering a few central variables in managing emergencies typical of complex systems. In summary, the results of the analysis conducted highlight the importance of proactively addressing the challenges associated with emergency decision-making during disasters by promoting better communication, increased staff training, and the development of emotional and cognitive skills necessary to deal with critical situations effectively.

## 5. Conclusions

The growing interest in research led to the question of whether there were any studies that investigated decision-making processes in emergency management with reference to institutional decision-makers in such a way as to find and list useful data for future guidance. The present systematic review of the literature on the subject led to the selection of nine studies that highlighted crucial issues in terms of operational practices employed by institutional decision-makers in emergency contexts. The fundamental assumption is that, while there are several successes in terms of emergency management, there are also considerable failures due to difficulties attributable to variables on which preventive action can be taken. But this is not always possible. One of the basic considerations in this area is the computational limits of human cognition. Managing a lot of problematic information for the decision-maker does not help the human cognitive system to faithfully represent the situations to be faced [67]. Importance is placed by these studies on the centrality of communication in emergencies and at the decision-making level. Communication exchanges by decision-makers in emergencies must be clear, fluid, and effective. Decisions can only be made based on available, reliable, and accurate information, but the selected studies have shown that, on several occasions, decisions are made with incomplete information. Information and data must be able to be shared and used interactively throughout the decision-making chain. What role does emotionality and emotional competence play in institutional decision-makers? How can one intervene by increasing emotional awareness during the management of an emergency?

Time pressure combined with the scarcity of information often generates strong emotions and, sometimes, stress in institutional decision-makers. It would be desirable to study these emotional aspects in an ecological environment in the future, despite all the difficulties of conducting research in emergencies. The key question that emerged regarding the concept of training emergency managers with a specific focus on decision-making issues is as follows: “What is the best form of training for institutional decision-makers so that they have the necessary skills to deal adequately with emergencies?” The complex nature of decision-making in emergency scenarios does not facilitate traditional training models but emphasizes the need to tailor training provision to the type of realistic scenario in which institutional decision-makers in emergencies are often placed, characterized by rapid choices, tight time constraints, and information gaps. Dynamism is the key attribute in CPS; the variables at play are constantly changing; meanwhile, the urgency linked to emergencies requires continuous decisions, and it is difficult for the decision-maker to relate and connect the most disparate variables [12]. In these cases, problems related to the limited resources of the human cognitive system emerge once again [11]; and it is precisely in these cases that humans have equipped themselves with methods and tools capable of supporting their decisions. One of these is the chain of command and control (C2). The command-and-control chain (C2) during emergencies becomes a fundamental element for managing complex problems [37]. The optimization of responses to critical events required the organization into work teams to better deal with emergencies. These teams operate under the precepts of command and control (C2) [38].

Future investigations into the relationship between and typologies of the chain of command and control during real emergencies might better illuminate the issue of centralization of decisions versus decentralization of decisions. This hope might help those decision-makers who have pointed out as a problem the lack of clarity regarding coordination responsibilities and poor coordination of responses during crises. These factors, in fact, have been mentioned as a possible obstacle to the natural flow of operations in emergencies. Drawing attention to the contemporary scenario increasingly driven by technological decision support, we feel compelled to urge future research questions to investigate how technological decision support systems are employed in complex problem-solving and dynamic decision-making during emergency management in institutional settings to support decision-makers. The strategic objective of this research aims to be a useful support for experts in the field to effectively facilitate coping with adverse emergency events that occur without warning. It is hoped that these questions will guide future research to enrich the body of knowledge in this area.

## Figures and Tables

**Table 1:** Results of quality assessment of the studies data.

Studies	1.	2.	3.	4.	5.	6. *	7. *	8. **	9.	10. ***	11.	12.	13. ****	14.	Quality Rating
[54]	Y	Y	Y	Y	Y	Y	CD	NA	Y	NA	Y	NR	NA	Y	Good
[55]	Y	Y	N	Y	Y	N	Y	NA	Y	NA	Y	N	NA	N	Fair
[56]	Y	Y	NR	NA	N	N	Y	NA	Y	NA	Y	NR	NA	N	Poor
[57]	Y	N	N	Y	N	N	Y	NA	Y	NA	Y	NR	NA	N	Fair
[58]	Y	Y	Y	Y	Y	N	Y	NA	Y	NA	Y	NR	NA	Y	Good
[59]	Y	N	NA	Y	N	N	NA	NA	Y	NA	Y	N	NA	N	Good
[60]	Y	Y	Y	Y	NR	N	NR	NA	Y	NA	Y	NR	NA	N	Fair
[61]	Y	Y	Y	Y	N	N	Y	NA	Y	NA	Y	NR	NA	N	Fair
[62]	Y	N	NR	N	N	N	CD	NA	Y	NA	NR	NR	NA	N	Very Poor
[63]	Y	Y	N	Y	N	N	Y	NA	Y	NA	N	NR	NA	N	Fair

Note: Quality of included studies was assessed using the National Institutes of Health (NIH) Quality Assessment tool for Observational Cohort and Cross-Sectional Studies (https://www.nhlbi.nih.gov/health-topics/study-quality-assessment-tools, accessed on 28 September 2023). 1. Was the research question or objective in this paper clearly stated? 2. Was the study population clearly specified and defined? 3. Was the participation rate of eligible persons at least 50%? 4. Were all the subjects selected or recruited from the same or similar populations (including the same period)? Were inclusion and exclusion criteria for being in the study prespecified and applied uniformly to all participants? 5. Was a sample size justification, power description, or variance and effect estimates provided? 6. For the analyses in this paper, were the exposure(s) of interest measured before the outcome(s) being measured? 7. Was the timeframe sufficient so that one could reasonably expect to see an association between exposure and outcome if it existed? 8. For exposures that can vary in amount or level, did the study examine different levels of the exposure as related to the outcome (e.g., categories of exposure, or exposure measured as a continuous variable)? 9. Were the exposure measures (independent variables) clearly defined, valid, reliable, and implemented consistently across all study participants? 10. Was the exposure(s) assessed more than once over time? 11. Were the outcome measures (dependent variables) clearly defined, valid, reliable, and implemented consistently across all study participants? 12. Were the outcome assessors blinded to the exposure status of participants? 13. Was loss to follow-up after baseline 20% or less? 14. Were key potential confounding variables measured and adjusted statistically for their impact on the relationship between exposure(s) and outcome(s)? CD: cannot determine; NA: not applicable; NR: not reported; N: no; Y: yes; *: For cross-sectional analyses, the answer to Questions 6 and 7 should be “no”; **: If there are only two possible exposures (yes/no), then this question should be given an “NA”, and it should not count negatively towards the quality rating; ***: Cross-sectional studies do not assess the exposure(s) more than once because of their nature; ****: Cross-sectional studies do not require a follow-up.

**Table 2:** EDM Data.

Studies	Authors	Country	Sample	Assessment of EDM
			N	
[54]	Terry Oroszy, 2018	USA-UK-RSA- IR- TR	15	AdHoc_Q
[55]	Roberts and Wernstendt, 2018	USA	231 (19 Females)	AdHoc_Q
[56]	Celik and Corbacioglu, 2018	Turkey	58	Semi-Structured Interview
[57]	Hoekstra and Montz, 2017	USA	23	Semi-Structured Interview
[58]	Dilawar et al., 2019	Pakistan	268 (28 Females)	GDMS Inventory
[59]	Al-Dabbagh, 2020	Iraq	15	Semi-Structured Interview
[60]	Paton et al., 2018	New Zeland	30	Semi-Structured Interview
[61]	Peerbolte and Collins, 2013	USA	54 (16 Females)	WGCTA-S
[63]	Wilkinson et al., 2021	UK	185 (47 Females)	AdHoc_Q

Note: USA: United States of America; RSA: Republic of South Africa; IR: Iran; TR: Turkey; AdHoc Q: Ad Hoc Questionnaire; GDMS Inventory: General Decision-Making Style Inventory; WGCTA-S: Watson Glaser Critical Thinking Appraisal Short Form.

## Data Availability

The data from this research can be requested at the following e-mail address: ivan.dalessio@uniroma1.it.
